# Context-Dependent Diversity-Effects of Seaweed Consumption on Coral Reefs in Kenya

**DOI:** 10.1371/journal.pone.0144204

**Published:** 2015-12-16

**Authors:** Austin T. Humphries, Christopher D. McQuaid, Tim R. McClanahan

**Affiliations:** 1 Coastal Research Group, Department of Zoology and Entomology, Rhodes University, Grahamstown, South Africa; 2 Coral Reef Conservation Project, Wildlife Conservation Society, Mombasa, Kenya; 3 Marine Programs, Wildlife Conservation Society, Bronx, New York, United States of America; University of Otago, NEW ZEALAND

## Abstract

Consumers and prey diversity, their interactions, and subsequent effects on ecosystem function are important for ecological processes but not well understood in high diversity ecosystems such as coral reefs. Consequently, we tested the potential for diversity-effects with a series of surveys and experiments evaluating the influence of browsing herbivores on macroalgae in Kenya’s fringing reef ecosystem. We surveyed sites and undertook experiments in reefs subject to three levels of human fishing influence: open access fished reefs, small and recently established community-managed marine reserves, and larger, older government-managed marine reserves. Older marine reserves had a greater overall diversity of herbivores and browsers but this was not clearly associated with reduced macroalgal diversity or abundance. Experiments studying succession on hard substrata also found no effects of consumer diversity. Instead, overall browser abundance of either sea urchins or fishes was correlated with declines in macroalgal cover. An exception was that the absence of a key fish browser genus, *Naso*, which was correlated with the persistence of *Sargassum* in a marine reserve. Algal selectivity assays showed that macroalgae were consumed at variable rates, a product of strong species-specific feeding and low overlap in the selectivity of browsing fishes. We conclude that the effects of browser and herbivore diversity are less than the influences of key species, whose impacts emerge in different contexts that are influenced by fisheries management. Consequently, identifying key herbivore species and managing to protect them may assist protecting reef functions.

## Introduction

Herbivory is an important process that promotes coral reef health through removal of algae [1–3]. When herbivores are removed from coral reefs, ecological niches are potentially left unoccupied [4, 5]. In these scenarios, algae may outcompete corals for access to light and space [6], creating a system dominated by foliose macroalgae that reflect diverse structural, chemical, and nutritional characteristics [7]. With increasing anthropogenic stressors, it is important to examine under what conditions, how quickly, and in what ways reefs may transition into and out of less desirable forms to avoid reef states that support fewer ecosystem services and have negative feedbacks [8, 9].

Herbivore communities on coral reefs are shaped by multiple physical and biological factors, which change through space and time. Fishing, however, has been shown to influence herbivore community structure heavily through selective removal of species or functional groups [10]. On coral reefs in Kenya, for example, fishing pressure influences the balance between sea urchins and fishes and creates two distinct consumer groups with associated foraging behaviors [11, 12]. Here, sea urchins are spatially constrained herbivores and typically consume algal turfs and macroalgae intensely on a small spatial scale [13]. In contrast, herbivorous fishes are vagile and exhibit greater feeding selectivity in their diet [14]. As a result, the succession and persistence of fleshy macroalgae may be dependent on the type and life histories of the herbivores present [15]. Consequently, emerging trends in fisheries management are aimed at promoting different groups of herbivores, including trap modifications that reduce capture of certain species, or temporary spatial closures that allow some recovery of herbivores [16–18]. Assessing how fisheries management influences herbivores and their ability to remove algae is important for decisions that concern species, functional groups, or overall diversity.

Browsing herbivores are important in removing established macroalgae and are heavily fished in the Kenyan coral reef fishery [McClanahan and Mangi 2004]. While scraping and grazing herbivores forage on algal turf, they may also remove macroalgal recruits from within the epilithic turf matrix and be expected to influence macroalgal diversity [19]. Some studies attribute species diversity as a driving mechanism [4, 20, 21], whereas others stress the importance of a few key species [20, 22–24]. Regardless, many of the studies informing these discussions are from Australia’s Great Barrier Reef (GBR) [4, 22, 25, 26]. It is still relatively unclear, however, how well these results transfer to reefs with different stressors. For example, the socioeconomic context of many countries in the Western Indian Ocean region results in heavy fishing on coral reefs, whereas the opposite is true for the GBR where large-scale environmental processes may be more important. Such location-specific differences among coral reef systems make it important to evaluate the consequences of changing herbivore assemblages in the appropriate context.

While knowledge of functional differences among coral reefs herbivores has increased greatly [2, 27, 28], the interaction of herbivore feeding and fishing and management options is still poorly understood. Therefore, this study was undertaken to evaluate how fishing and management impact functional and diversity effects of herbivores for macroalgal removal and persistence. To do this, we combined underwater visual census data with a manipulative field experiment in three different management systems representing a gradient of fishing influence. First, using survey data, we tested the hypothesis that herbivore diversity would be strongly associated with reduced algal abundance and community composition on reefs. Then, we used a manipulative feeding experiment to study responses of macroalgae in the different fisheries management systems. These data aim to provide a better understanding of how local-level fisheries management influences the effects of consumer diversity on coral reefs in Kenya, particularly in the case of browsing herbivores feeding on macroalgae.

## Materials and Methods

Kenya’s Office of the President provided clearance to do research in Kenya, Kenya Wildlife Service provided permission to work in the government closures, and Kuruwitu Community Organization and Mradi/Kanamai Fishers BMU provided permission to work in their community closures.

There was no collection of any vertebrate organisms for this study, therefore no approval was needed or obtained by an Institutional Animal Care and Use Committee or equivalent animal ethics committee. Algae were collected as part of the experiment and all sampling procedures and manipulations were reviewed or specifically approved as part of obtaining the field permit.

### Study sites

This study was conducted in 2011 and 2012 along a ~150 km stretch of coast in Kenya at sites in back-reef lagoons that are protected from strong waves. We chose sites representing distinct fisheries management regimes: two heavily fished open access reefs, two younger, small community managed marine reserves, and two older, large government reserves (S1 Fig). We regarded management regimes as experimental treatments and sites representing these treatments were interspersed. The community closures, Kuruwitu and Mradi, have received protection from fishing since 2005 and 2010, respectively, and are both approximately 0.3–0.4 km^2^ in size. The government closures, Mombasa and Malindi, have received protection from fishing since 1991 and 1968, respectively; Mombasa is approximately 6 km^2^ in size, whereas Malindi is 10 km^2^. Compliance in the community and government closures is high (Humphries personal observation). Fishing at Kanamai and Ras Iwatine, which are unprotected, is intense and highly unselective with a variety of gear types being used (e.g. spear guns, nets, traps), while beach seines were not used at Ras Iwatine. Kenya has a ~4 m tidal range, but the sites are shallow at low tides, typically less than 1.5 m. Humphries et al. [15] showed that fish and sea urchin assemblages, as well as benthic compositions, differed significantly with fisheries management, allowing us to use management regime for comparisons in this study. See Humphries et al. [15] for detailed tables and statistical tests, but to summarize, the greatest coral to macroalgae ratio is at Mradi, a community closure, and Kanamai, an open access reef. Ras Iwatine, another open access reef, had the lowest coral cover, whereas Mombasa, a government closure, had the highest macroalagal abundance.

### Field surveys of herbivores and benthic communities

To characterize existing grazer communities and substratum at all sites, sea urchins and fishes were sampled concurrently with benthic cover using quadrats and belt transects. Sampling was conducted during neap tides when the water was between ~1 and 4 m deep. Sea urchins were identified to the species-level and counted in 10 m^2^, haphazardly placed plots (n = 9–18 per site). Wet weight was estimated by multiplying average numbers of individuals by average wet weights per species [11]. Fish were counted and size (total length, TL) estimated to the nearest 10 cm at the family level by underwater visual census (via snorkel) using 2–4 replicate belt transects (5 x 100 m) per site. Herbivorous fishes were counted separately and size estimated to the nearest 5 cm TL at the species level along the same transects. Parrotfishes (subfamily Scarinae) < 10 cm were grouped together as ‘juveniles’ due to the difficulty of identifying these to species in the field. Mass was determined by converting fish counts to biomass with published length-weight relationships [29, 30] and assigned to feeding groups (scrapers, grazers, browsers) based on published information on diets [30, 31]. Benthic cover was surveyed using 9 haphazardly placed 10 m line-intercept transects. The distances covered by each major benthic component (hard and soft coral, turf algae, crustose coralline algae [CCA], and erect macroalgae) underlying each transect line were measured to the nearest centimeter. Corals and macroalgae were further identified to the genus level and percentage cover was calculated as the sum of the lengths divided by the total transect length.

We created a Bray-Curtis similarity matrix for each site and used non-metric multidimensional scaling (MDS) ordination to visualize similarities in herbivore community structure across sites. We also used linear regression to determine whether herbivore diversity (Shannon’s *H*), herbivore species richness, browser fish and sea urchin biomass (kg ha^-1^) is correlated with existing macroalgal abundance (% cover) and diversity.

### Macroalgal removal experiment

To assess the efficacy of herbivores in browsing previously established macroalgae, we allowed algae to grow on experimental substrata (plates) protected by cages (1 x 1 x 0.5 m, L x W x H) for over one year (~390 days; Aug 2011 to Sept 2012) at all six study sites before allowing access by herbivores. This amount of time was chosen to allow macroalgae the opportunity to fully develop into a late stage of succession and mimic substratum that had gone from algal turfs to dominance by large macroalgae. Cages were made from plastic mesh material (2.5 x 2.5 cm square holes) and attached to bare substratum using u-bolts. Previous work found that cages similar to these had no significant effect on algal standing crop or species composition other than the effect of excluding grazers [32]. Experimental substrata were made from ~2.5 cm cross-sections of dead massive *Porites* coral (mean size [± SE] was 177 ± 15 cm^2^, n = 96) and the plates had flat surfaces but irregularly shaped edges. Holes were drilled in individual plates, which were attached to plastic cage flooring at least 5 cm apart in groups of four. Each replicate per treatment comprised one of these sets of 4 plates. Treatments (n = 4 replicates site^-1^) were placed > 20 m apart from one another at each of the six study sites (S2 Fig).

After cages were removed, all experimental plates were left in situ and exposed to herbivores for ~90 d (Sept to Nov 2012). Description of the algal community present on the plates was achieved by taking digital photographs every ~14 d. Photographs were processed and percentage composition of algal turf, fleshy algae, calcareous algae, and encrusting coralline algae (CCA) was determined using a random point-intercept method (50 points per plate) with digital photography software (Adobe Photoshop). Fleshy algae were further identified to the genus level as *Sargassum*, *Padina*, *Hypnea*, *Dictyota*, *Turbinaria*, or *Cystoseira*. All other macroalgal genera were grouped as ‘Other’ and never comprised more than 5% of total cover. Values for percentage cover were determined for each plate and then averaged for that replicate.

To determine if percentage cover of total macroalgae (all fleshy algae combined), as well as cover of individual macroalgal genera, changed from the start of the experiment to the final sampling event, we used separate one-way ANOVAs at each site. Data were log-transformed when necessary to achieve normality and alleviate heterogeneity of variances among the data. Model diagnostics were performed visually using frequency histograms, funnel and Q-Q plots, and the final models (using raw data or log-transformed data) met the assumptions of normality and homogeneity of residuals.

To determine how algal community structure changed through time on the experimental substrata, we performed a multivariate randomization procedure. The macroalgal abundance data were used as factors to create a Bray-Curtis similarity matrix for each site at the initial and final sampling events. Each (site) matrix was then analyzed using permutational analysis of variance (PERMANOVA), which analyzes distance measures in a linear model with categorical variables to test for significance of factors (n = 999 permutations). Factors used in the PERMANOVA were the different groupings, or genera, of algae (e.g. algal turf, *Dictyota*, *Hypnea*, *Sargassum*). Pairwise tests among factors were analyzed to determine if and when site effects caused significant differences in algal community structure. We then used MDS ordination to visualize similarities in algal community structure across sites and over time. Ordination showed the presence of contrasting algal communities between the government closure sites, Mombasa and Malindi, at the final sampling event. Therefore, to investigate fish species correlated with these differences, we ran a similarity of percentages (SIMPER) analysis using a subset of the fish species matrix (only herbivores).

### Feeding selectivity assays

To determine feeding selectivity of browsing herbivorous fishes, we collected six common macroalgal genera from fished reefs and deployed them within a deeper channel (~4 m) in Mradi, a community-managed fisheries closure. This site was chosen at it contained a wide diversity of herbivore species typically observed on Kenyan reef lagoons. We used underwater digital video cameras (video length was approximately 4 h) and quantified loss of algal mass relative to caged controls over a 24 h period. Macroalgae selected for the experiment were: *Sargassum*, *Dictyota*, *Hypnea*, *Padina*, *Cystoseiria*, and *Turbinaria*. These six macroalgal genera were chosen because they are among the most commonly found macroalgae on reefs along the coast of Kenya [15], encompass a wide range of morphological and functional forms, and represent the Rhodophyta and Phaeophyta taxonomic divisions. However, individual algae were chosen and care taken to ensure that the same morphospecies was used within each genus (e.g. algae thalli phenotypically similar), as well as to reflect common sizes of each genus found on Kenyan reefs.

Each specimen was visually inspected to roughly standardize size among individuals within genera. Excess water was removed from each alga using a salad spinner (10 revolutions) and the plant was then weighed in the field to the nearest 0.01g. Initial masses of macroalgal genera were (g; mean ± SE): *Sargassum* sp. (1.79 ± 0.08), *Dictyota* sp. (0.89 ± 0.07), *Hypnea* sp. (0.90 ± 0.07), *Padina* sp. (0.95 ± 0.05), *Cystoseira* sp. (1.17 ± 0.08), and *Turbinaria* sp. (1.28 ± 0.07). Algae were then transported in plastic self-sealed bags and one thallus of each of the six macroalgal genera was attached in random order approximately 10 cm apart from one another on an 80 cm section of nylon rope. Each alga was held in place using a clothespin attached to the rope and weighed down using 200g weights. Paired treatment (exposed to herbivores; n = 5) and control (caged; n = 5) ropes were assembled in the same manner. Cages for control lines were made from plastic mesh material (2.5 x 2.5 cm square holes) and placed over assays to prevent browsing by all large-bodied herbivores [32].

Each experimental trial began by placing paired treatment and control ropes along the reef in areas dominated by coral and accessible to herbivores during both low (~1 m depth) and high (~4 m depth) tidal periods. Paired treatment and control ropes were attached to dead coral fragments within 1 m of one another, and deployed on the substratum at 10–15 m intervals. Assays were deployed at calm periods between 0800–1000 h during neap tidal cycles. The experiment was not run during spring tidal cycles because of the strong currents associated with the ~4 m tidal range in Kenya. Assays were collected after 24 h and weighed as for initial mass. Mass consumed, *M*_*c*_, by herbivores was calculated as

$$M_{c}=\left[T_{i}*\left(\frac{C_{f}}{C_{i}}\right)-T_{f}\right]$$ (1)

where *T*_*i*_ and *T*_*f*_ were the initial and final masses (g), respectively, of a treatment alga exposed to herbivores, and *C*_*i*_ and *C*_*f*_ were the initial and final masses (g), respectively, of its paired control. Percentage consumed was calculated to allow comparisons among macroalgal genera. Thirteen trials were performed during three separate neap tidal cycles in the calm, northeast monsoon season (August and September) in 2012, each with five paired treatment and control assay lines that were randomly moved along the reef prior to each trial.

Herbivore feeding preferences and rates of grazing on macroalgae may depend on the identity and quantity of other available resources. When multiple macroalgae (i.e. treatments) are simultaneously offered to herbivores in feeding assays, they may not be independent and therefore violate the assumptions of ANOVA procedures [33]. Consequently, to test for differences in the percentage of macroalgal mass consumed by herbivores, we used a rank-based Friedman’s test and significant differences were further evaluated using Friedman’s post-hoc multiple comparison tests [34]. For these models, we averaged results for each fish species within a trial and used each trial as an independent replicate (n = 13 replicates).

Stationary underwater video cameras (GoPro; n = 2) were used during each trial to identify herbivores feeding on the macroalgal assays. Cameras were deployed at the onset of each trial (0800–1000 h) and positioned 1–2 m from a haphazardly selected treatment rope. After deployment, filming continued without disturbance for ~2.5 h. In total, 26 treatment assays were filmed (~64 h) over the 13 replicate trials. All video footage was viewed and the number of bites taken on the experimental assays by each species of fish was counted and scaled (bites h^-1^). Videoed feeding assays were conducted on different treatment ropes concurrently within a replicate trial, and therefore may not be independent, so we averaged results for each fish species within a trial (n = 13 replicates). To determine if feeding rates differed among macroalgae for each of the six dominant browsing herbivores, we used separate rank-based Friedman’s tests with significant differences followed by Friedman’s post-hoc multiple comparison tests [34]. The six dominant herbivore fish species accounted for > 95% of all recorded bites on macroalgae. To characterize the herbivorous fish community, visual census methods were used as described above and performed above assay lines at the time of deployment. All data analyses were performed using the program R, version 2.15.1 [35].

## Results

### Field surveys of herbivores and benthic communities

**Fig 1:**
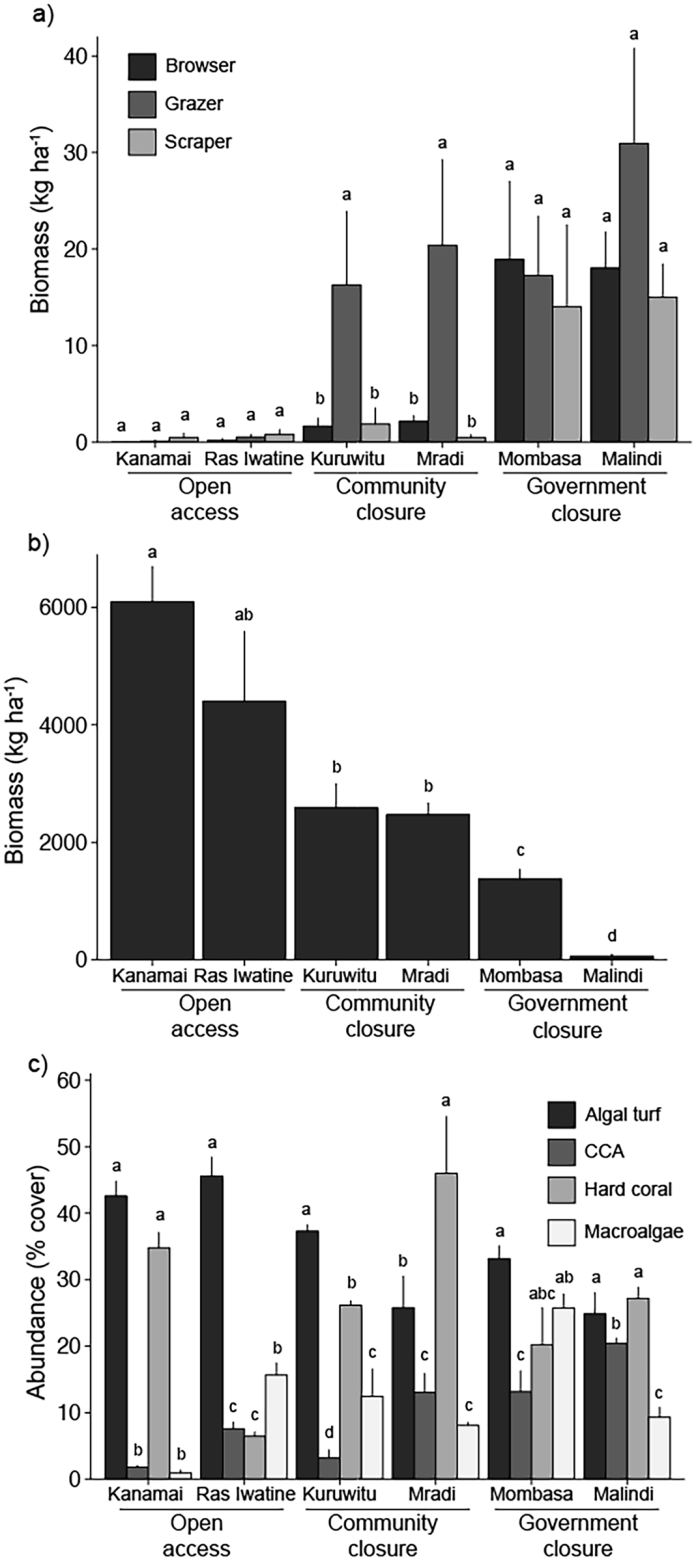
Mean (± SE) (a) biomass of herbivorous fishes (kg ha-1), (b) biomass of sea urchins (kg ha-1), and (c) benthic composition (% cover). Letters represent homogenous subgroups.

**Fig 2:**
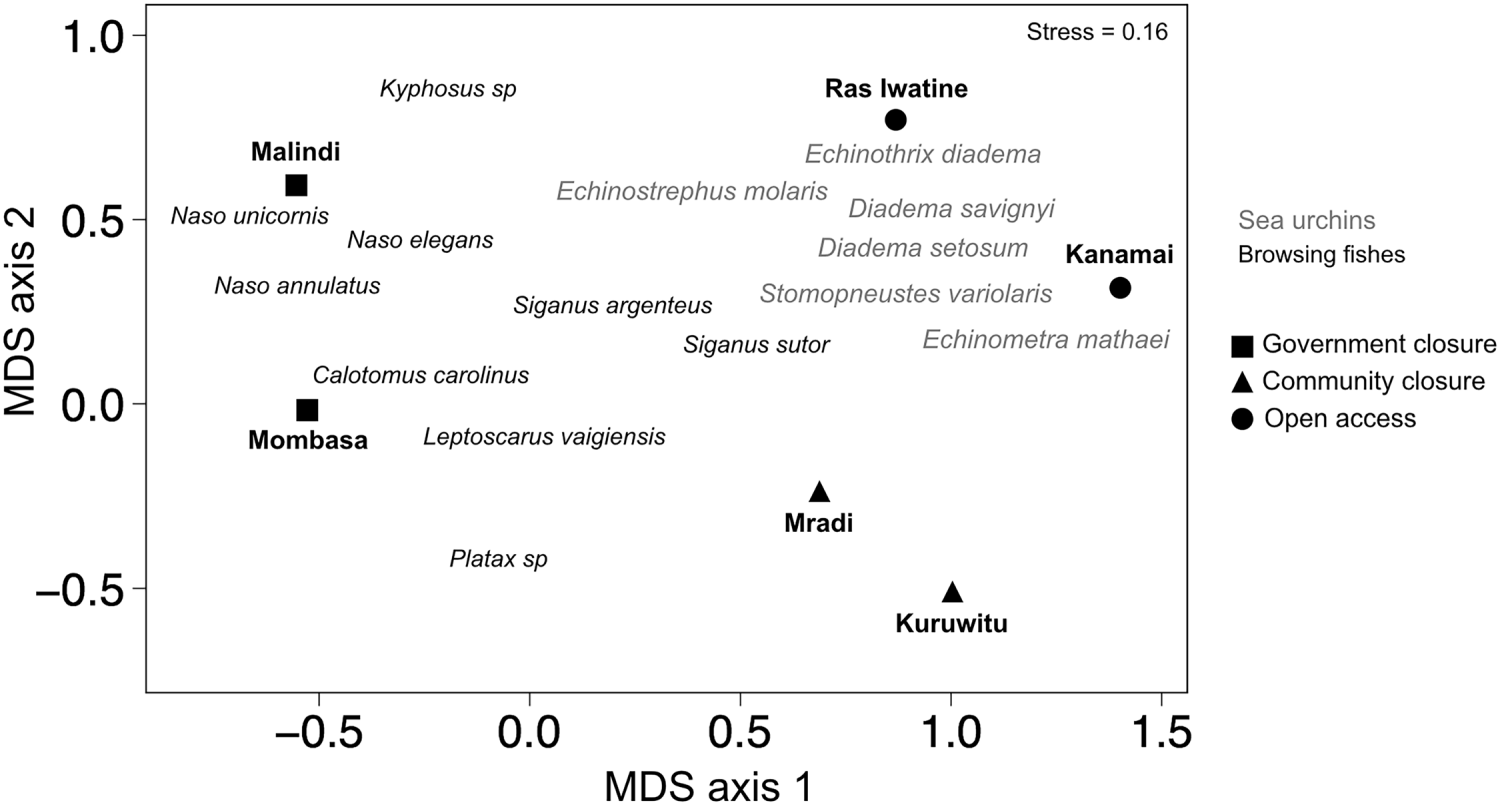
Non-metric multidimensional scaling analysis (MDS) of herbivorous fishes and sea urchin species abundance estimates at the six study sties. Shapes represent fisheries management regime. Points that are closer together in ordination space are more similar in terms of community composition, and individual browsing fishes (black) and sea urchins (grey) are overlaid to visualize dominant species.

There was a clear influence of human fishing pressure on herbivorous fish and sea urchin communities (Fig 1a and 1b). There were more sea urchins and fewer fishes at open access sites and vice versa at government closures (p < 0.05). Community closure sites had intermediate biomass of sea urchins as well as herbivorous fishes, but lacked browsing fishes (Fig 1a and 1b). The government closures differed primarily in browser fish composition, with more *Naso* species at Malindi (Fig 2, S1 Table).

Existing benthic cover varied among sites with the highest hard coral and lowest macroalgal cover at Mradi, a community closure, and Kanamai, an open access reef (Fig 1c, S1 Table). Ras Iwatine, an open access reef, had the lowest hard coral and Mombasa, a government closure, had the highest macroalgal abundance, while crustose coralline algae (CCA) was most abundant at Malindi, another government closure (Fig 1c, S1 Table). Algal turf abundance was highest at the open access reefs, Kanamai, and Ras Iwatine (Fig 1c, S1 Table).

**Fig 3:**
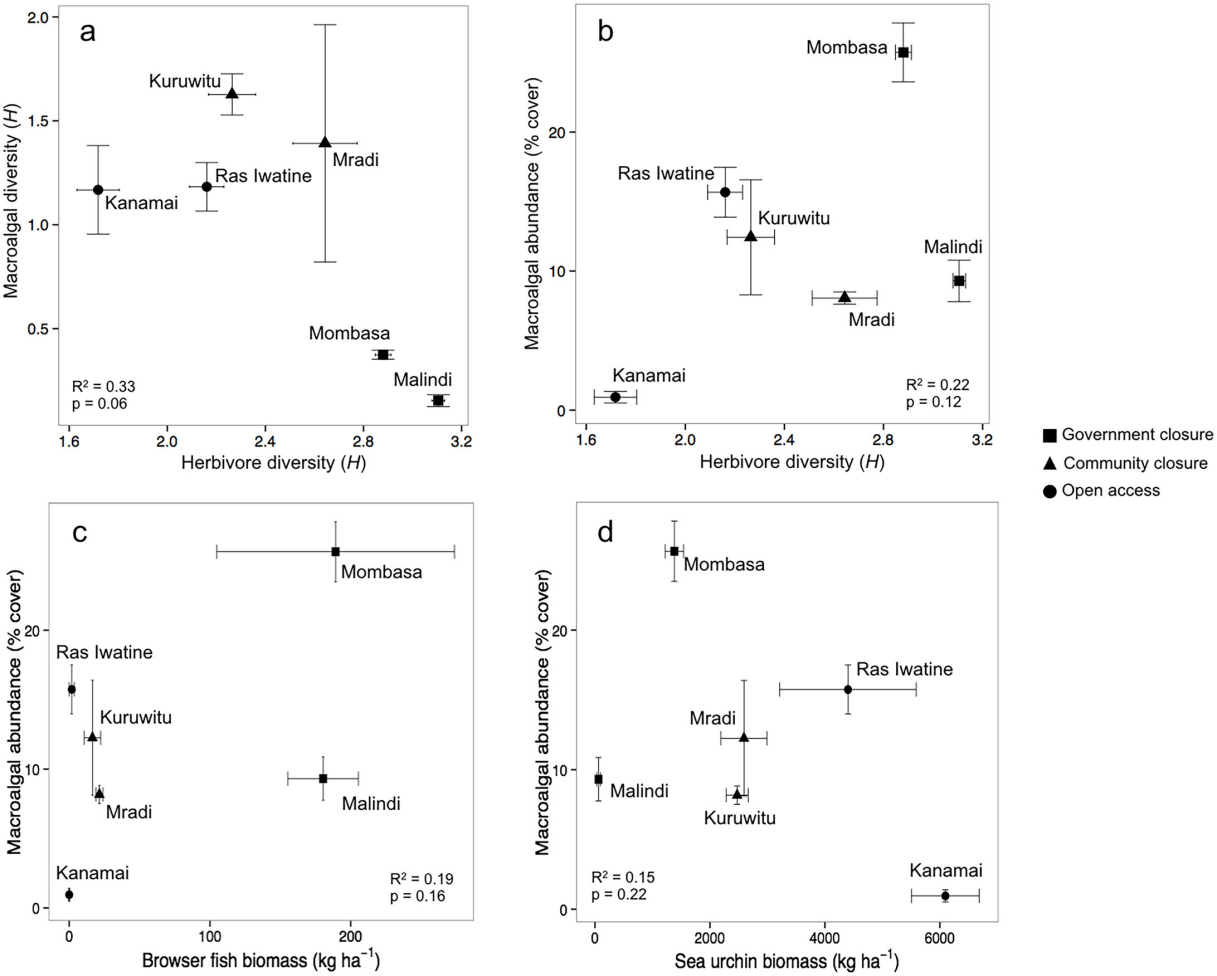
Relationships between (a) herbivore and macroalgal diversity (Shannon’s *H*) (x ± SE), (b) herbivore diversity and macroalgal abundance (% cover), (c) herbivorous fish biomass (kg ha^-1^) and macroalgal abundance, and (d) sea urchin biomass and macroalgal abundance at each of the six study sites. Shapes represent fisheries management at each site.

Herbivore fish and sea urchin diversity and species richness followed the restrictions on fishing with generally lower diversity and richness at open access reefs than in marine reserves, and highest in the old and larger government managed closures (S1 Table). The diversity (and richness) of herbivores displayed a non-significant relationship with both macroalgal diversity and abundance (Fig 3a and 3b). The biomass of fish browsers was greatest in government closures and was not significantly related to macroalgal abundance (Fig 3c). Sea urchin biomass was lowest at government closures and also not significantly related to macroalgal abundance (Fig 3d).

### Macroalgal removal experiment

**Fig 4:**
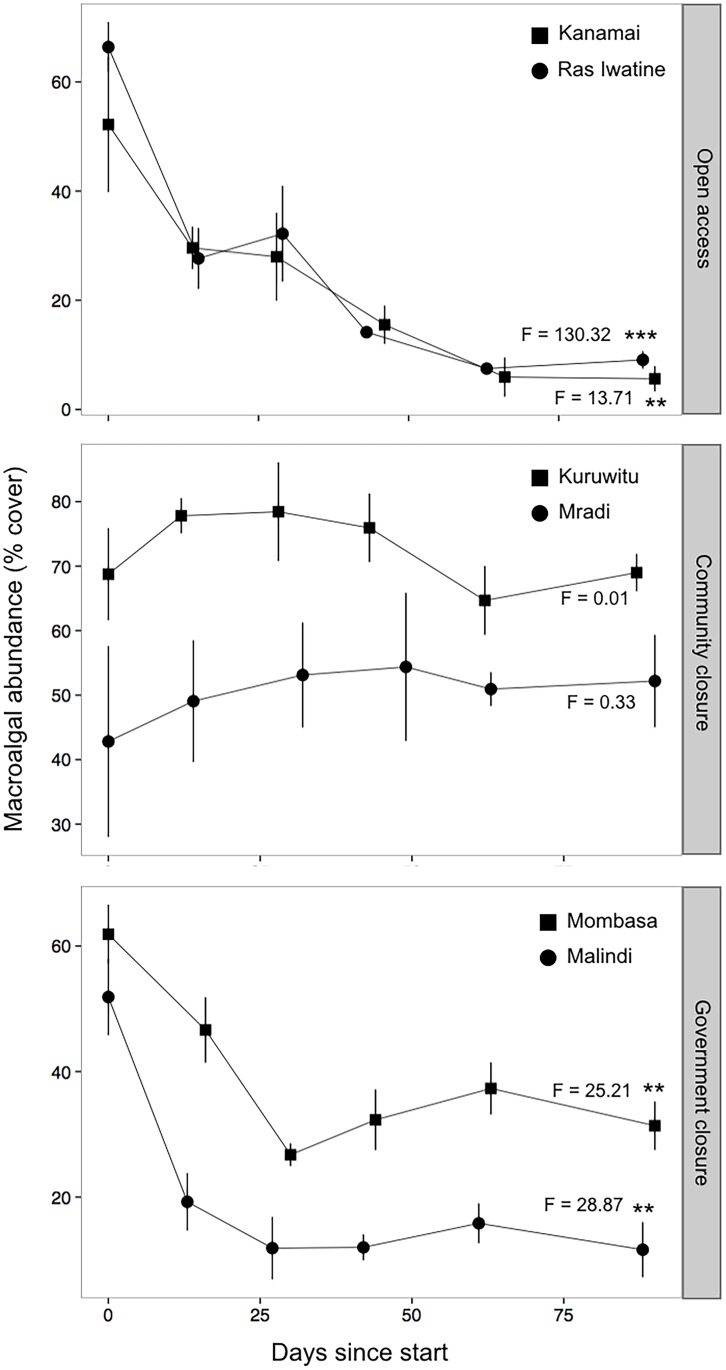
Time series of the mean abundance (% cover ± SE) of total macroalgae on the experimental substrata at the six study sites over the ~90 day experimental period, presented by the three fisheries management categories. Algae grew on plates for over one year before removing cages and exposing them to herbivory. Note different scales on y-axes. Asterisks indicate significant differences in abundance from the beginning of the experiment to the final sampling event at each site (d.f. = 1). ***p<0.001, **p<0.01, *p<0.05.

After exposure of the experimental substrata to herbivores, macroalgal cover declined significantly (p < 0.05) in government closures and open access sites but not community closures (Fig 4, S3 Fig). In the open access and government closure sites, macroalgal abundance was reduced from initial levels of 50–70% to 25% and lower. Changes in algal composition on the experimental substrata varied among sites over the ~ 90 day experiment (S3 Fig). In the two open access reefs, Kanamai and Ras Iwatine, cover of *Dictyota* and *Sargassum* was reduced significantly (p < 0.05) and all other macroalgal genera were at least partially reduced with the exceptions of *Turbinaria* and *Hypnea* at Kanamai (Table 1). Macroalgal cover changed little in community closures, with the exception of a significant decline in *Padina* at Kuruwitu. In contrast, *Sargassum* cover increased significantly at Kuruwitu, whereas other macroalgal genera either increased or decreased only slightly (and not significantly). At Mombasa and Malindi, the government closures, all macroalgal genera cover declined, except *Sargassum* at Mombasa and *Turbinaria* at Malindi, both of which increased in cover over the ~90 day period.

**Table 1:** Results of one-way ANOVAs used to evaluate abundance of macroalgal genera on experimental substrata, from the beginning of the experiment to the final sampling event. An overall increase in macroalgal abundance is indicated by a ‘+’ symbol and a decrease by a ‘-’ symbol.

	Open access						Community closure						Government closure					
	Kanamai			Ras Iwatine			Kuruwitu			Mradi			Mombasa			Malindi		
	F-value	p-value	+ / -	F-value	p-value	+ / -	F-value	p-value	+ / -	F-value	p-value	+ / -	F-value	p-value	+ / -	F-value	p-value	+ / -
*Dictyota*	8.98	*	-	713.20	***	-	0.04	0.855	NA	1.00	0.356	NA	1.04	0.346	NA	NA	NA	NA
*Hypnea*	0.82	0.400	NA	24.96	**	-	0.16	0.700	NA	3.04	0.132	NA	1.60	0.253	NA	1.23	0.310	NA
*Padina*	2.34	0.177	NA	4.90	0.069	NA	32.14	***	-	0.22	0.652	NA	3.93	0.095	NA	7.86	*	-
*Sargassum*	11.43	*	-	19.33	**	-	25.58	**	+	0.69	0.438	NA	0.11	0.757	NA	10.75	**	-
*Turbinaria*	1.00	0.356	NA	1.00	0.356	NA	NA	NA	NA	NA	NA	NA	NA	NA	NA	0.81	0.403	NA
*Cystoseira*	5.60	0.056	NA	0.41	0.548	NA	1.00	0.356	NA	0.48	0.516	NA	107.14	***	-	1.90	0.217	NA

Asterisks indicate significant results.

***p<0.001,

**p<0.01,

*p<0.05.

**Fig 5:**
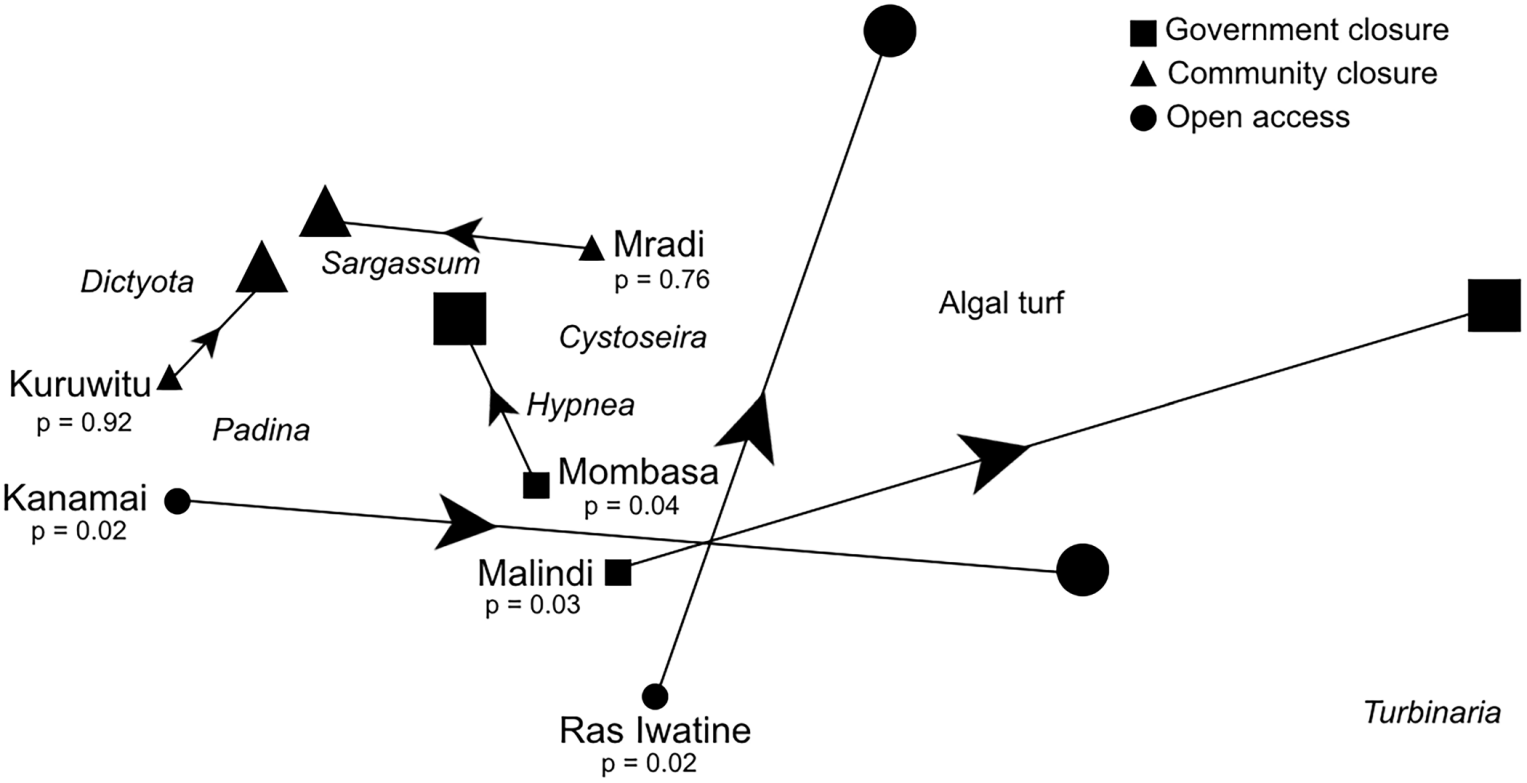
Non-metric multidimensional scaling analysis (MDS) of successional trajectories showing similarity of algal communities in the six study sites on the experimental plates after cages were removed to the final sampling event (~90 days). Fisheries management regimes indicated by different symbols next to the initial algal communities on day 0 and the final 90 day samples are the enlarged data points. Arrows indicate direction of change over time and are scaled to the magnitude of change in the algal community. Distance matrices were analyzed separately for each site using permutational analysis of variance (PERMANOVA; n = 999 permutations; Stress = 0.11).

Algal composition at the community closures and one government closure site, Mombasa, converged towards a similar *Sargassum* dominance by the end of 90 days despite the different algal communities at the beginning of the experiment (Fig 5). Nevertheless, the shifts in composition were minor compared to the large changes in two fished sites, Kanamai and Ras Iwatine, and the unfished Malindi site. After ~ 90 days these sites were dominated by algal turf. SIMPER analysis indicated that the unicornfishes, *Naso elegans* (18.9%) and *N*. *unicornis* (17.5%), as well as the sea chub, *Kyphosus vaigienesis* (16.1%), contributed most to dissimilarities between the browser fish communities at the two government closure sites. Other species contributing to at least 70% difference between Mombasa and Malindi were the two species of rabbitfishes, *Siganus argenteus* (13.9%) and *S*. *sutor* (11.8%).

### Feeding selectivity assays

**Fig 6:**
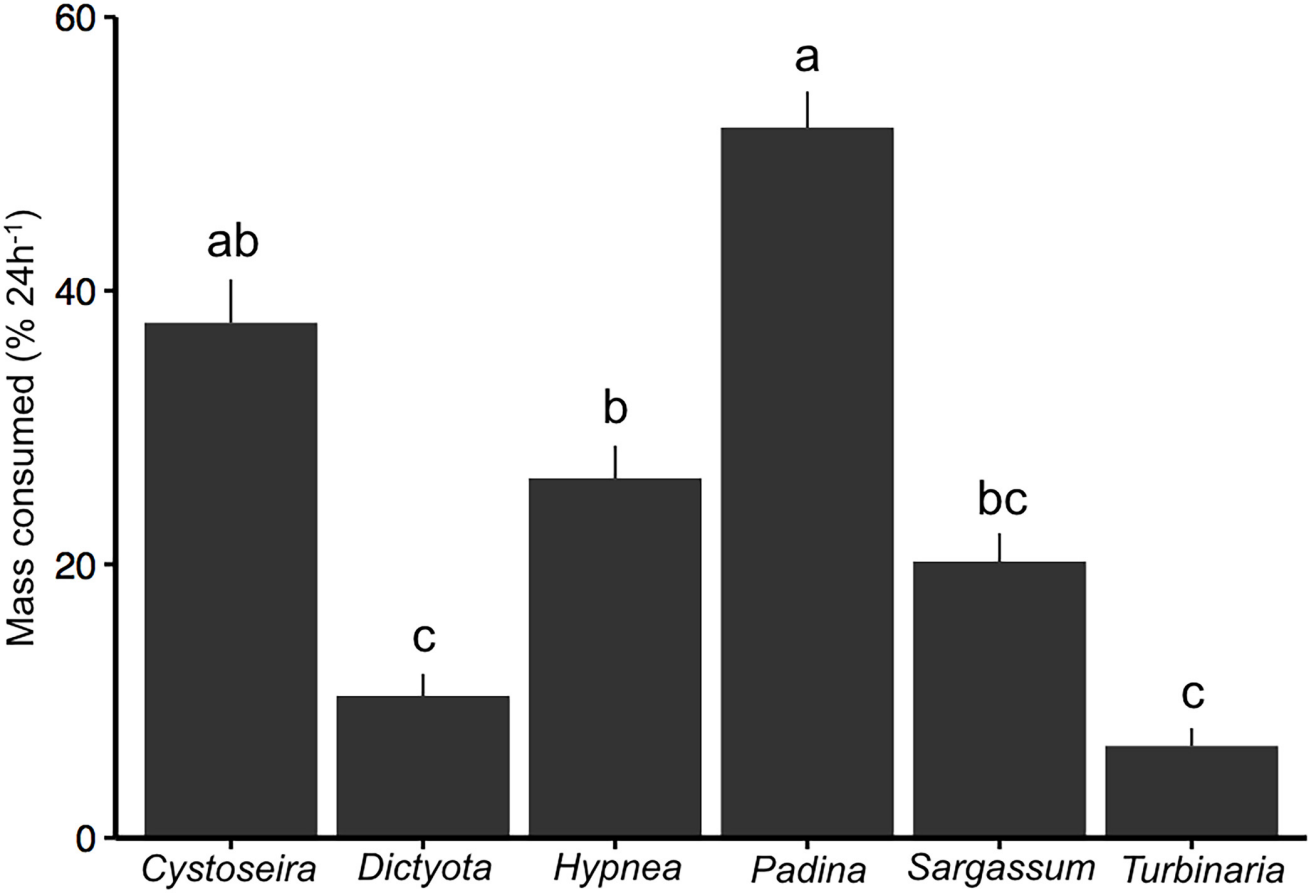
Mass of macroalgae removed by herbivorous fishes (% 24h^-1^; x ± SE, n = 13) for 6 taxa of algae used in selectivity assay experiments within the Mradi community closure. Letters indicate homogeneous subgroups.

**Fig 7:**
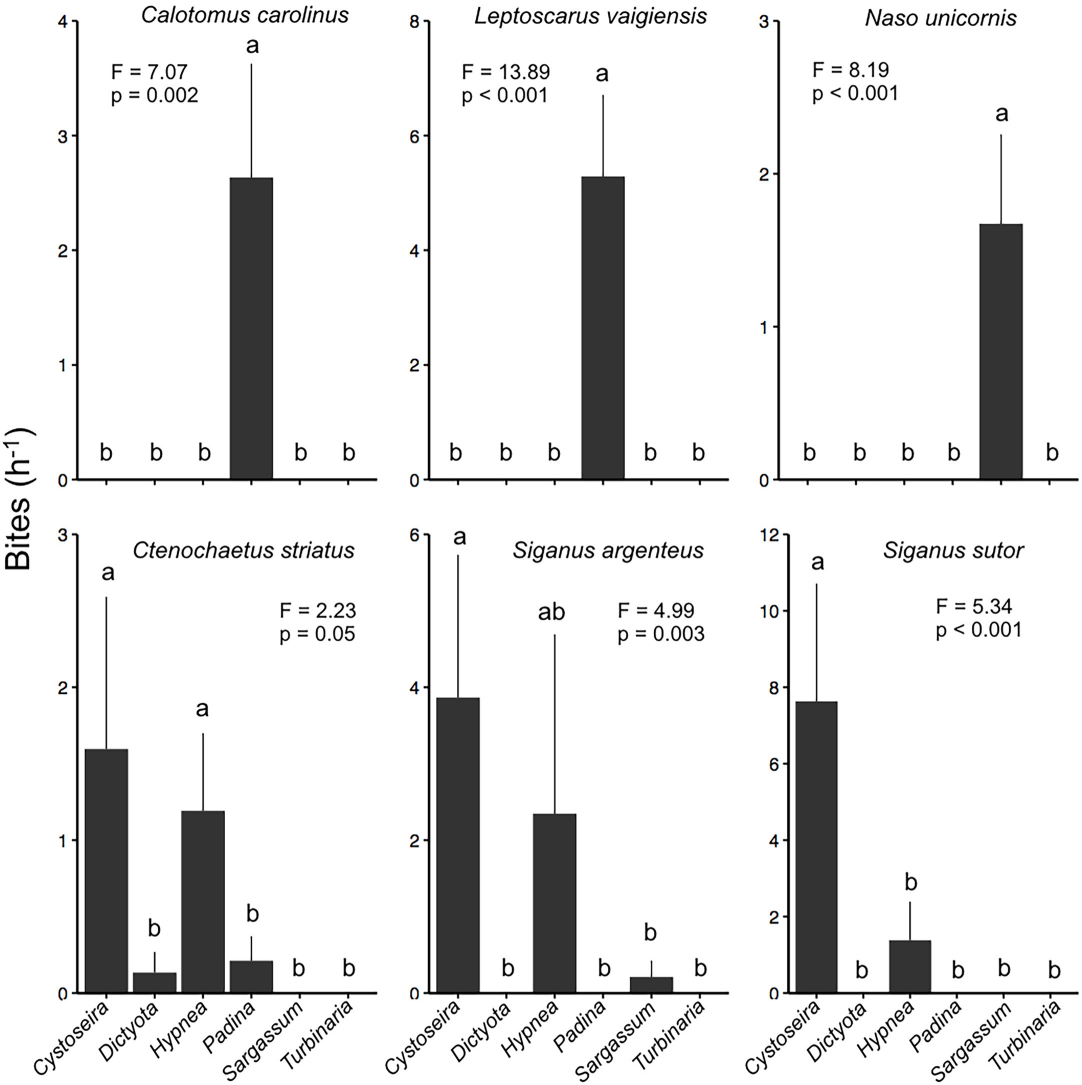
Bite rate of browsing fishes (h^-1^; x ± SE, n = 13) on experimental selectivity assays of macroalgae in Mradi (in the channel) community closure. Letters indicate homogeneous subgroups. Note scale differences between y-axes.

Biomass of herbivorous fishes was 460.8 kg ha^-1^ where the feeding selectivity assay experiment was performed (Mradi channel; S2 Table). Browser species that feed primarily on macroalgae accounted for 32% of the overall herbivorous fish biomass and the grazing surgeonfish *Acanthurus nigrofuscus* was the most abundant herbivore (81.9 kg ha^-1^). *Padina* was consumed at a greater rate (51.7% 24h^-1^) in the multiple-choice feeding assays than all other genera except Cystoseira (Fig 6). *Dictyota* and *Turbinaria* were consumed significantly less than *Padina*, *Hypnea*, and *Cystoseira*. We recorded a total of 1,472 fish bites on the six experimental genera and a clear algal preference for *Padina*, *Cystoseira*, or *Hypnea* was observed (Fig 7). The parrotfishes *Calotomus carolinus* and *Leptoscarus vaigienesis* fed exclusively on *Padina*, whereas the unicornfish *Naso unicornis* fed exclusively on *Sargassum*. Both species of rabbitfishes *Siganus sutor* and *S*. *argenteus* fed mostly on *Cystoseira*, with some feeding on *Hypnea*. The surgeonfish *Ctenochaetus striatus* fed mostly on *Cystoseira* and *Hypnea*, but also some *Dictyota* and *Padina*. Video observations revealed, however, that consumption of these macroalgae was rare, as surgeonfish were observed spitting out the algae.

## Discussion

Despite clear evidence of the effects of browsing herbivores in algal assays and reductions of algae in experimental transplants, there was no clear relationship between herbivore diversity and existing macroalgal diversity or abundance on these reefs. Government-managed marine reserves did have a greater diversity of herbivores that was correlated with reduced macroalgal diversity but not with macroalgal abundance due largely to abundance of some highly herbivore resistant taxa, such as *Turbinaria*. No consumer diversity-effects were observed in the experimental algal plates study. Nevertheless, macroalgal cover declined in reefs with high browser abundances, dominated by either sea urchins in open access fished reefs or fishes in old and large no-take marine reserves. One exception was where the absence or low numbers of a key browser genus, *Naso*, correlated with the persistence of *Sargassum* in Mombasa, a government-managed marine reserve. The assay experiment indicated strong species-specific feeding and low diet overlap among browsing fishes. Consequently, feeding preferences drove interactions between a few key browsing fish species, which play a critical role in the consumption of seaweeds, especially in high diversity reefs with few sea urchins. These findings corroborate similar results from other studies in Australia, Fiji, and the Caribbean, providing geographic breadth for herbivore diversity-effects and feeding influences on macroalgae [3, 4, 14, 20, 23, 26, 36].

Macroalgae are common on reefs following disturbances or removal of fish biomass through fishing [37–39]. Further, sea urchins and fishes are expected to affect algae communities differently [13, 15, 40]. In this study, we found complementary feeding and species-specificity of feeding to be high among the browsing fish community but we found the opposite among the sea urchin community; diversity-effects in this study were least important on fished reefs dominated by sea urchins. Macroalgae at fished reefs were, as predicted, consumed non-selectively and reduced significantly despite low sea urchin diversity and differences in the species composition of sea urchin assemblages (*Echinometra mathaei* was the dominant sea urchin species at Kanamai, and *Echinothrix diadema* at Ras Iwatine). Thus, sea urchins appear to be less affected by competitive interactions or chemical and structural defenses in algae and appeared to be non-selective foragers in our system as predicted more broadly [41–43]. Non-selective foraging has also been found in temperate ecosystems where sea urchin abundance is high [44]. This finding may be especially important for understanding impacts and management needs in the Caribbean where sea urchins have been significantly reduced from historic levels and fishes are the dominant herbivores [45, 46].

Site and taxon-specific roles of herbivore guilds in each fisheries management category led to variable responses of macroalgae on the experimental substrata. For example, *Sargassum* largely dominated assemblages at the community closures where its cover did not change after the cage-removal. This result was correlated with the almost complete absence of browsing fishes and a lower abundance and impact of the diverse sea urchin community. At open access fished reefs, high abundances of sea urchins (> 4400 kg ha^-1^) was correlated with the suppression of all types of macroalgae. Seaweed abundance plummeted to ~10% cover, even though the diversity of sea urchins was low. Browsing fishes had similar correlations with macroalgae at the government closure sites where most fleshy algal taxa were quickly reduced. One exception was at Mombasa reserve where *Sargassum* remained prevalent throughout the experiment (~30% cover) even though browser diversity was high; *Sargassum* was not consumed due to the absence of a few key unicornfishes, primarily *Naso unicornis* and *N*. *elegans*, and perhaps the sea chub *Kyphosus vaigiensis* [21]. Additionally, *Turbinaria* increased slightly throughout the experiment at Malindi, suggesting that, once established, it may persist even in the presence of high fish abundance. While these findings show clear correlations with herbivores, there is a possibility that some macroalgal loss was due to environmental factors such as waves. However, this experiment was conducted in the northeast monsoon season, which is characterized by low wind energy [47] and a previous study by Humphries et al. [15] indicates that macroalgal losses from flow or wave exposure are negligible in this system.

The observed absence of a relationship between herbivore diversity and macroalgae provides an explanation for why some marine reserves fail to recover to coral-dominance once macroalgae are established [39, 48]. For instance, Mombasa has a high diversity of herbivores, comprising mostly fishes, but lacked a subset of unicornfishes (*Naso* sp.), which appear to be particularly important for the removal of *Sargassum*. Therefore, other species of macroalgae were reduced while *Sargassum* remained prevalent (~23% cover). Here, diversity-effects were dependent on *Naso unicornis*, which has also been shown to be an important consumer of *Sargassum* in Fiji, the Great Barrier Reefs, and Ningaloo [4, 20, 21, 23, 49], as well as on degraded reefs in the Seychelles [48]. On the other hand, Malindi had a lower diversity of herbivores but included a portfolio of key species, including grazers and scrapers that feed in a complementary fashion to one another (Humphries et al. 2014), reducing both the diversity and abundance of algae on the experimental plates. The differences in browsing fish assemblages may be driven by the existing benthic composition [48], where diet preferences are mediated by available resource quality and quantity. These results support accumulating studies from other regions indicating the importance of maintaining herbivores with complementary feeding strategies to increase reef resilience [3, 4, 20, 21, 23, 36].

Most coral reef feeding studies have found that maintaining the numbers and complementarity of herbivores is critical for maintaining the low algal cover that is necessary for effective maintenance of the reef calcifying function [3, 14, 20]. Our findings corroborate these findings but emphasize that only a few species that represent a small portion of the overall herbivorous fish community can account for most of the feeding on macroalgae [20]. *Naso unicornis*, for example, was responsible for 100% of the browsing on *Sargassum*, but represented only 4% of the herbivorous fish abundance and biomass. Similarly, the parrotfishes *Calotomus carolinus* and *Leptoscarus vaigiensis* represented 12% of the herbivorous fish abundance and biomass, but accounted for 96% of *Padina* consumption. The absence of one of these browsing fish species may lead to the proliferation of their preferred food, though perhaps with the relaxation of competitive interactions, other species will change their feeding habits [50]. We do, however, also acknowledge the limitations of an assay that was conducted at a single study site, such as the potential effects of relative consumer size and abundance on selection [51].

Diet partitioning and selectivity among parrotfishes here did differ from reports in other reefs. For example, the parrotfishes observed by Mantyka and Bellwood [14] on the GBR in Australia, and Rasher et al. [20] in Fiji, were of the genera *Hipposcarus*, *Chlorurus*, and *Scarus*, and fed on different species of macroalgae with varying degrees of selectivity. In the GBR, they fed on calcareous algae, *Halimeda* sp., [14], and in Fiji, on the red algae *Galaxaura* and *Amphiroa*, as well as algal turf [20]. Such differences may be a function of the species found on these and other reefs, and their respective feeding modes [24] as they represent a different evolutionary lineage (Scarinae) to those in this Kenyan study. Scarinae are primarily regarded as grazers of algal turf, but under experimental conditions may also feed on macroalgae [52]. Scarinae have jaw morphologies that allow them to scrape the substratum and even take deeper excavating bites from hard surfaces [53]. In contrast, the parrotfishes we observed feeding on macroalgae were of the genera *Calotomus* and *Leptoscarus*, and are not known to feed on algal turf [54], have teeth that allow clipping of algae [25, 55], and are from the Sparisomatinae evolutionary lineage. We observed these species feeding exclusively on the brown algae *Padina*.

## Conclusions

Herbivores that consume macroalgae are critical in performing key ecological processes and facilitating the health of reef-building corals [4, 22]. Our findings indicate that the removal of macroalgae is not strictly dependent upon herbivore abundance or diversity, but that browser identity is particularly important through complementary feeding, especially where sea urchins are absent, as in no-take marine reserves. Consequently, using diversity as a key resilience indicator for coral reefs may be an over-simplified management approach [56], where species-specific feeding characteristics could override diversity-effects [3, 20, 36]. This demonstrates the importance of feeding selectivity and complementarity on coral reefs, and also highlights the potential for low functional redundancy even where species diversity is high [57]. No-take closures in heavily fished seascapes may not be reaching conservation targets because of slow recovery or absence of key species [15, 58], and here we show that the consequences may be crucial for the proliferation of macroalgae and the maintenance of coral reef states and processes. While these results may not be novel in the context of similar studies from other regions of the world [3, 4, 14, 20, 23, 26, 36], they do provide confirmatory data from a new region of the world and provide consensus in support of managing for a portfolio of browsing and grazing herbivores with unique and complementary roles (e.g. specific browsers, as well as grazers and scrapers) to promote reef resilience.

## Supporting Information

S1 Fig Schematic of experimental design. (DOCX)

S2 Fig Time series of the mean percentage cover (with SE) of individual macroalgal genera. (DOCX)

S3 Fig Time series of the mean percentage cover (with SE) of individual macroalgal genera on the experimental coral plates at the six study sites over a ~90 day period. Shapes indicate macroalgal genera. Algae were allowed to grow on plates with no herbivory (in cages) for over one year before starting the experiment. Notice different scales on y-axes.
(DOCX)

S1 Table Mean (± SE) biomass (kg ha^-1^) of (a) herbivorous fishes and (b) sea urchins at the six study sites, as well as (c) mean abundance (% cover ± SE) of the substratum, organized by fisheries management. (DOCX)

S2 Table Mean herbivorous fish biomass (kg ha^-1^ with SE) at Mradi (in the channel) community closure, where selectivity assays were performed. (DOCX)
