# Supplementary figures and images for: Context-Dependent Diversity-Effects of Seaweed Consumption on Coral Reefs in Kenya
Source: PLoS One. 2015 Dec 16;10(12):e0144204. doi: 10.1371/journal.pone.0144204 (PMC4684473; doi:10.1371/journal.pone.0144204)

**S1 Fig.** Study sites with marine closure sizes in parentheses.

**
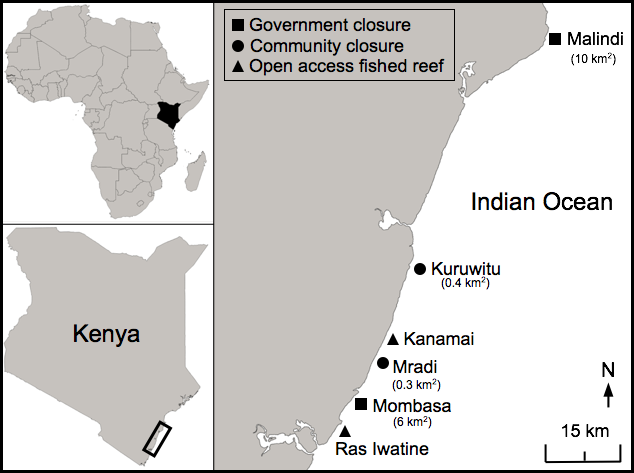
**

Supplement: S1 Fig — (DOCX) [file pone.0144204.s001.docx]
