# Supplementary material for: Context-Dependent Diversity-Effects of Seaweed Consumption on Coral Reefs in Kenya
Source: PLoS One. 2015 Dec 16;10(12):e0144204. doi: 10.1371/journal.pone.0144204 (PMC4684473; doi:10.1371/journal.pone.0144204)
Supplement: S2 Fig — (DOCX) [file pone.0144204.s002.docx]

**S2 Fig.** Schematic of experimental design for substrata testing the efficacy of browsing herbivores in removing previously established macroalgae. Algae were allowed to grow on experimental substrata for > 1 y before being subjected to herbivory.
