# Supplementary material for: Context-Dependent Diversity-Effects of Seaweed Consumption on Coral Reefs in Kenya
Source: PLoS One. 2015 Dec 16;10(12):e0144204. doi: 10.1371/journal.pone.0144204 (PMC4684473; doi:10.1371/journal.pone.0144204)
Supplement: S3 Fig — Shapes indicate macroalgal genera. Algae were allowed to grow on plates with no herbivory (in cages) for over one year before starting the experiment. Notice different scales on y-axes. (DOCX) [file pone.0144204.s003.docx]

**S3 Fig.** Time series of the mean percentage cover (with SE) of individual macroalgal genera on the experimental coral plates at the six study sites over a ~90 day period. Shapes indicate macroalgal genera. Algae were allowed to grow on plates with no herbivory (in cages) for over one year before starting the experiment. Notice different scales on y-axes.
