# Supplementary material for: Context-Dependent Diversity-Effects of Seaweed Consumption on Coral Reefs in Kenya
Source: PLoS One. 2015 Dec 16;10(12):e0144204. doi: 10.1371/journal.pone.0144204 (PMC4684473; doi:10.1371/journal.pone.0144204)
Supplement: S1 Table — (DOCX) [file pone.0144204.s004.docx]

**S1 Table.** Mean (± SE) biomass (kg ha^-1^) of (a) herbivorous fishes and (b) sea urchins at the six study sites, as well as (c) mean abundance (% cover ± SE) of the substratum, organized by fisheries management.

(a)

| Fishes |  |  | Open access |  |  | Community closure | |  | Government closure | |
| --- | --- | --- | --- | --- | --- | --- | --- | --- | --- | --- |
| Family | Species | Functional group | Kanamai | Ras Iwatine |  | Kuruwitu | Mradi |  | Mombasa | Malindi |
| Acanthuridae | *Ctenochaetus striatus* | Grazer | 0 (0) | 3.7 (0.1) |  | 89.1 (8.5) | 105.2 (2.6) |  | 39.8 (4.5) | 123.2 (9.8) |
|  | *Acanthurus nigrofuscus* | Grazer | 0 (0) | 0.9 (0.9) |  | 0 (0) | 67.7 (4.1) |  | 38.8 (38.8) | 56.1 (22.8) |
|  | *Zebrasoma scopas* | Grazer | 0 (0) | 0 (0) |  | 0 (0) | 1.5 (1.5) |  | 56.4 (21.1) | 26.9 (17.4) |
|  | *Naso annulatus* | Browser | 0 (0) | 0 (0) |  | 0 (0) | 2.5 (2.5) |  | 3.5 (3.5) | 22.5 (10.2) |
|  | *Acanthurus triostegus* | Grazer | 0.9 (0.9) | 0 (0) |  | 57.3 (2.1) | 0 (0) |  | 0 (0) | 0 (0) |
|  | *Zebrasoma veliferum* | Grazer | 0 (0) | 0 (0) |  | 0 (0) | 0 (0) |  | 5.5 (5.5) | 0 (0) |
|  | *Acanthurus leucosternon* | Grazer | 0 (0) | 0 (0) |  | 0 (0) | 7.1 (7.1) |  | 9.1 (2) | 55.3 (15.9) |
|  | *Ctenochaetus strigosus* | Grazer | 0 (0) | 0 (0) |  | 0 (0) | 2 (2) |  | 0 (0) | 0 (0) |
|  | *Acanthurus nigricauda* | Grazer | 0 (0) | 0 (0) |  | 0 (0) | 0 (0) |  | 0 (0) | 7.3 (2.6) |
|  | *Acanthurus dussumieri* | Grazer | 0 (0) | 0 (0) |  | 0 (0) | 0 (0) |  | 5.5 (5.5) | 9.7 (0.2) |
|  | *Naso elegans* | Browser | 0 (0) | 0 (0) |  | 0 (0) | 0 (0) |  | 0 (0) | 15.8 (2.8) |
|  | *Naso unicornis* | Browser | 0 (0) | 0 (0) |  | 0 (0) | 0 (0) |  | 0 (0) | 10.5 (6.8) |
| Labridae | *Scarus sordidus* | Scraper | 0 (0) | 0 (0) |  | 13.5 (13.5) | 2.1 (2.1) |  | 95.1 (25.5) | 29.7 (2.6) |
|  | *Scarus ghobban* | Scraper | 0 (0) | 3.5 (2.1) |  | 0 (0) | 0 (0) |  | 0 (0) | 29.2 (11.2) |
|  | *Calotomus carolinus* | Browser | 0 (0) | 0 (0) |  | 0 (0) | 5.6 (1.4) |  | 92.2 (46.9) | 46.4 (10.2) |
|  | *Scarus frenatus* | Scraper | 0 (0) | 0 (0) |  | 0 (0) | 0 (0) |  | 0 (0) | 9 (9) |
|  | *Scarus psittacus* | Scraper | 0 (0) | 2.8 (2.8) |  | 0 (0) | 0 (0) |  | 0 (0) | 12.6 (3.6) |
|  | *Scarus niger* | Scraper | 0 (0) | 0 (0) |  | 0 (0) | 0 (0) |  | 0 (0) | 16.6 (7.6) |
|  | *Scarus rubroviolaceus* | Scraper | 0 (0) | 0 (0) |  | 0 (0) | 0 (0) |  | 0 (0) | 22.1 (14) |
|  | *Hipposcarus harid* | Scraper | 0 (0) | 0 (0) |  | 0 (0) | 0 (0) |  | 15.1 (15.1) | 0 (0) |
|  | *Leptoscarus vaigiensis* | Browser | 0 (0) | 0 (0) |  | 7.8 (0.8) | 3.5 (3.5) |  | 24.9 (9.9) | 26.1 (10) |
|  | Juvenile sp (< 10cm) | Scraper | 3.7 (3.3) | 0.2 (0.1) |  | 1.4 (1) | 1.6 (0.5) |  | 2 (0.9) | 0.8 (0.5) |
| Pomacanthidae | *Centropyge multispinis* | Grazer | 0 (0) | 0 (0) |  | 0 (0) | 4.9 (1.4) |  | 6.3 (3.2) | 7.4 (1.9) |
| Siganidae | *Siganus sutor* | Browser | 0 (0) | 1.8 (1.8) |  | 8.7 (5) | 2.8 (0.9) |  | 0 (0) | 24 (24) |
|  | *Siganus argenteus* | Browser | 0 (0) | 0 (0) |  | 0 (0) | 0 (0) |  | 61.2 (26.9) | 15 (15) |
| Ephippidae | *Platax teira* | Browser | 0 (0) | 0 (0) |  | 0 (0) | 2.1 (2.1) |  | 1.1 (1.1) | 0 (0) |
| Kyphosidae | *Kyphosus vaigiensis* | Browser | 0 (0) | 0 (0) |  | 0 (0) | 0 (0) |  | 0 (0) | 9.7 (3.4) |
|  | Total fishes |  | 4.6 (4.2) | 12.9 (5.8) |  | 177.7 (24.7) | 208.6 (2) |  | 456.6 (44.7) | 578.9 (23.6) |

(b)

| Sea urchins |  |  | Open access | |  | Community closure | |  | Government closure | |
| --- | --- | --- | --- | --- | --- | --- | --- | --- | --- | --- |
| Family | Species | Functional group | Kanamai | Ras Iwatine |  | Kuruwitu | Mradi |  | Mombasa | Malindi |
| Diadematidae | *Diadema savignyi* | Sea urchin | 562.5 (340.3) | 841.2 (33.8) |  | 534.7 (34.7) | 645.8 (48.6) |  | 20.8 (6.9) | 20.8 (6.9) |
|  | *Diadema setosum* | Sea urchin | 541.7 (375) | 441.2 (307.8) | | 816.7 (166.7) | 179.2 (37.5) |  | 0 (0) | 16.7 (16.7) |
|  | *Echinothrix calamaris* | Sea urchin | 0 (0) | 0 (0) |  | 0 (0) | 154.2 (38.6) |  | 38.6 (0) | 0 (0) |
|  | *Echinothrix diadema* | Sea urchin | 115.7 (38.6) | 2956.3 (912.8) | | 38.6 (38.6) | 1079.6 (192.8) | | 983.2 (19.3) | 19.3 (19.3) |
| Echinometridae | *Echinometra mathaei* | Sea urchin | 4530.2 (1116.5) | 68.2 (2.2) |  | 768.2 (474.8) | 352.9 (88.9) |  | 148.5 (42.2) | 1.8 (1.8) |
|  | *Echinostrephus molaris* | Sea urchin | 0 (0) | 2.9 (0.1) |  | 0 (0) | 0.2 (0.2) |  | 6 (1.8) | 1.8 (1) |
| Stomopneustidae | *Stomopneustes variolaris* | Sea urchin | 0 (0) | 11.7 (3.3) |  | 4.2 (4.2) | 6.3 (6.3) |  | 8.3 (8.3) | 0 (0) |
| Toxopneustidae | *Tripneustes gratilla* | Sea urchin | 345.2 (151.4) | 79.8 (5) |  | 417.8 (6.1) | 51.5 (27.2) |  | 163.5 (151.4) | 0 (0) |
|  | *Toxopneustes pileolus* | Sea urchin | 0 (0) | 0 (0) |  | 11.1 (11.1) | 2.8 (2.8) |  | 11.1 (0) | 0 (0) |
|  | Total sea urchins |  | 6095.2 (591.2) | 4401.1 (1187.5) | | 2591.2 (402.8) | 2472.4 (192) |  | 1380 (160.8) | 60.4 (29.9) |

(c)

| Substrate |  |  | Open access | |  | Community closure | |  | Government closure | |
| --- | --- | --- | --- | --- | --- | --- | --- | --- | --- | --- |
| Classification | Name | Functional group | Kanamai | Ras Iwatine |  | Kuruwitu | Mradi |  | Mombasa | Malindi |
| Benthos | Hard coral | Major substratum | 34.77 (6.79) | 7.1 (4.56) |  | 26.17 (8.38) | 46.01 (13.12) | | 20.23 (10.08) | 27.17 (14.77) |
|  | Algal turf | Major substratum | 42.6 (9.48) | 42.8 (12.77) |  | 37.33 (13) | 21.05 (16.59) | | 33.18 (12.6) | 24.86 (15.49) |
|  | Macroalgae | Major substratum | 0.93 (2.01) | 13.88 (11.62) | | 12.44 (7.82) | 8.5 (6.27) |  | 25.73 (7.95) | 9.3 (6.34) |
|  | CCA | Major substratum | 1.77 (2.06) | 8.56 (2.61) |  | 3.18 (2.47) | 10.21 (5.35) |  | 13.16 (6.17) | 20.44 (7.94) |
| Macroalgae | *Cystoseira* | Fleshy algae | 0 (0) | 0 (0) |  | 1.57 (1.24) | 0.25 (0.25) |  | 0 (0) | 0 (0) |
|  | *Dictyota* | Fleshy algae | 0.03 (0.05) | 1.25 (1.4) |  | 0.75 (0.54) | 1.21 (0.02) |  | 0.45 (0.35) | 0.09 (0.13) |
|  | *Hypnea* | Fleshy algae | 0.04 (0.06) | 1.35 (0.54) |  | 0.41 (0.37) | 3.62 (2.53) |  | 0 (0) | 0.1 (0) |
|  | *Padina* | Fleshy algae | 0.25 (0.16) | 3.63 (0.37) |  | 1 (0.81) | 1.24 (0.2) |  | 0.03 (0.04) | 0 (0) |
|  | *Sargassum* | Fleshy algae | 0.07 (0.1) | 7.11 (1.37) |  | 4.93 (1.37) | 0.67 (0.67) |  | 23.17 (2.66) | 0 (0) |
|  | *Turbinaria* | Fleshy algae | 0.5 (0.53) | 0.52 (0.37) |  | 1 (0.11) | 0.83 (0.83) |  | 1.95 (0.03) | 9.04 (2.24) |
