# Supplementary material for: Context-Dependent Diversity-Effects of Seaweed Consumption on Coral Reefs in Kenya
Source: PLoS One. 2015 Dec 16;10(12):e0144204. doi: 10.1371/journal.pone.0144204 (PMC4684473; doi:10.1371/journal.pone.0144204)
Supplement: S2 Table — (DOCX) [file pone.0144204.s005.docx]

**S2 Table.** Mean herbivorous fish biomass (kg ha^-1^ with SE) at Mradi (in the channel) community closure, where selectivity assays were performed.

| Family | Species | Function | Biomass |
| --- | --- | --- | --- |
| Acanthuridae | *Acanthurus dussumieri* | Grazer | 51.9 (4.5) |
| Acanthuridae | *Acanthurus leucosternon* | Grazer | 0.2 (0.2) |
| Acanthuridae | *Acanthurus nigricauda* | Grazer | 7.9 (1.5) |
| Acanthuridae | *Acanthurus nigrofuscus* | Grazer | 81.9 (15.7) |
| Acanthuridae | *Acanthurus tennenti* | Grazer | 9.2 (2.2) |
| Acanthuridae | *Acanthurus triostegus* | Grazer | 8 (1.8) |
| Acanthuridae | *Acanthurus xanthopterus* | Grazer | 4.8 (2.8) |
| Acanthuridae | *Ctenochaetus striatus* | Grazer | 60.3 (3.6) |
| Acanthuridae | *Ctenochaetus strigosus* | Grazer | 0.1 (0.1) |
| Acanthuridae | *Naso annulatus* | Browser | 19.8 (0.3) |
| Acanthuridae | *Naso brevirostris* | Browser | 7 (3) |
| Acanthuridae | *Naso unicornis* | Browser | 19.3 (6.4) |
| Acanthuridae | *Zebrasoma scopas* | Grazer | 2.2 (0) |
| Ephippidae | *Platax teira* | Browser | 3.4 (1.7) |
| Kyphosidae | *Kyphosus vaigiensis* | Browser | 11.1 (2.9) |
| Pomacanthidae | *Centropyge multispinis* | Grazer | 3.3 (3.3) |
| Labridae | *Calotomus carolinus* | Browser | 42.2 (4.2) |
| Labridae | *Cetoscarus bicolor* | Scraper | 1.6 (0) |
| Labridae | *Leptoscarus vaigiensis* | Browser | 14.8 (3.7) |
| Labridae | *Scarus frenatus* | Scraper | 10.6 (4.6) |
| Labridae | *Scarus ghobban* | Scraper | 52.1 (2.3) |
| Labridae | *Scarus psittacus* | Scraper | 1.1 (1.1) |
| Labridae | *Chlorurus sordidus* | Scraper | 17.8 (3.6) |
| Siganidae | *Siganus sutor* | Browser | 30.2 (4.8) |
